# Phonon-Dominated In-Plane Thermal Conductivity of Single-Flake Metallic Ti_3_C_2_T_*x*_ MXene

**DOI:** 10.1021/acs.nanolett.6c01850

**Published:** 2026-07-30

**Authors:** Max C. van Hemert, Hugh Ramsden, Stefano Ippolito, Sebin Varghese, Bohai Liu, Elvira Tornero Gasca, Miguel Muñoz Rojo, Hai I. Wang, Yury Gogotsi, Klaas-Jan Tielrooij

**Affiliations:** † Department of Applied Physics and Science Education, 3169 Eindhoven University of Technology, Den Dolech 2, Eindhoven, 5612 AZ, The Netherlands; ‡ A.J. Drexel Nanomaterials Institute and Department of Materials Science and Engineering, Drexel University, Philadelphia, Pennsylvania 19104, United States; § Energy2Devices, 16379 Instituto de Ciencia de Materiales de Madrid (ICMM), Calle Sor Juana Inés de la Cruz 3, Madrid, 28049, Spain; ∥ Department of Thermal and Fluid Engineering, Faculty of Engineering Technology, University of Twente, Enschede, 7500 AE, The Netherlands; ⊥ Max Planck Institute for Polymer Research, Ackermannweg 10, Mainz, 55128, Germany; # Debye Institute for Nanomaterials Science, Utrecht University, Princetonplein 1, Utrecht, 3584, The Netherlands; 7 Catalan Institute of Nanoscience and Nanotechnology (ICN2), CSIC and BIST, Campus UAB, Bellaterra, Barcelona 08193, Spain

**Keywords:** MXene, heat transport, 2D materials, optothermal, spatiotemporal microscopy, Ti_3_C_2_T_*x*_

## Abstract

MXenes are a promising class of 2D materials, with prospects in applications such as electromagnetic interference shielding, energy storage, catalysis, and thermal management. Despite the importance of understanding their thermal transport properties, it is not clear whether electrons or phonons dominate heat transport, and the experimentally obtained in-plane thermal conductivities vary substantially. Here, we address this by studying in-plane heat transport in single Ti_3_C_2_T_*x*_ MXene flakes using spatiotemporal microscopy, directly visualizing heat transport in space and time. We obtain a thermal diffusivity of 0.05 cm^2^ s^–1^, corresponding to an in-plane thermal conductivity of 13–16 W m^–1^ K^–1^. Using an upper limit for the electronic conductivity based on terahertz measurements, we find that electrons carry at most half of the heat. This shows that—despite their metallic nature—phonons play an important role in the heat transport of Ti_3_C_2_T_*x*_ MXene, providing an unconventional combination of heat and charge transport properties.

## Graphical abstract

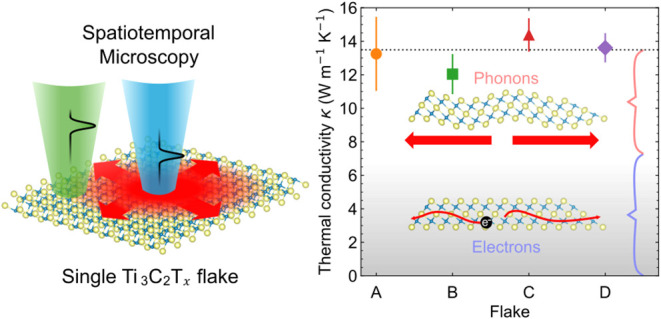

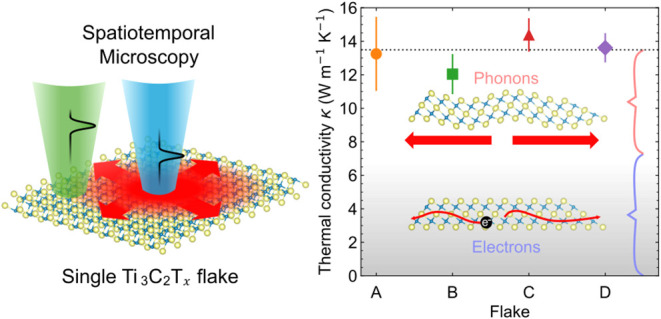

MXenes are a family of layered materials comprising molecularly thin layers of transition-metal carbides, nitrides, or carbonitrides, which are typically terminated by hydrophilic surface groups.^1,2^ Examples are titanium nitride, Ti_4_N_3_T_*x*_, molybdenum carbide, Mo_2_CT_*x*_, and titanium carbide, Ti_3_C_2_T_*x*_, where T_*x*_ represents mixed-termination groups, typically O, Cl, F, or OH. These materials are receiving increasing attention due to their interesting fundamental physical properties and their potential in a wide range of applications,^3,4^ including energy storage,^5,6^ thermal management,^7^ electromagnetic interference shielding,^8^ and sensing,^9,10^ while offering scalable synthesis routes, such as top-down etching and chemical vapor deposition.^4,11,12^ For these materials to see widespread use, it is important to understand their fundamental properties, including their thermal conductivity.^13^

There are currently two important open questions regarding in-plane heat transport of the most-studied and practically important MXene Ti_3_C_2_T_*x*_. First, the fundamental question of whether electrons or phonons dominate in-plane heat transport remains unanswered. Since this material has a metallic band structure,^14–16^ and heat conduction in metals typically occurs via electrons, electronic heat transport is expected to dominate over phononic heat transport. However, some previous studies point at phonon-dominated heat transport,^17,18^ while others point at electron-dominated transport.^19^ Second, the experimentally reported values for the in-plane thermal conductivity of Ti_3_C_2_T_*x*_ vary significantly: from below 1 W m^–1^ K^–1^ to 55 W m^–1^ K^–1^.^17–23^ An explanation for these inconsistent values may be that most previous studies have focused on a thin film geometry with many partially overlapping flakes.^17,18,20^ This creates challenges in probing intrinsic transport properties, as these measurements are sensitive not only to the intrinsic thermal conductivity but also to other factors, such as the flake size and thickness, the interflake thermal resistance,^17^ temperature,^24^ and the environmental humidity.^21^ Furthermore, MXenes are predicted to exhibit anisotropic thermal conductivity,^25,26^ which means that an experimental method is required that enables unambiguous decoupling of in- and out-of-plane thermal transport.

Here, we address these issues by studying the in-plane heat transport of individual Ti_3_C_2_T_*x*_ MXene flakes using spatiotemporal differential reflection microscopy. As illustrated in Figure 1a, spatiotemporal microscopy^27–30^ allows us to follow diffusion directly in space and time by examining the spatial broadening of an initial localized photoexcitation, shown in Figure 1b. In this way, we exclusively observe the in-plane diffusion of heat. This is a local technique where the initial optically pumped region has a diameter of around one micrometer, which is smaller than the typical flake size. Using an optical probe, we resolve transport-induced spatial broadening with a precision below 10 nanometers. This allows us to study individual Ti_3_C_2_T_*x*_ flakes and therefore obtain intrinsic heat diffusion properties. Moreover, because we directly observe heat transport in space and time, we obtain the thermal diffusivity without the need for prior knowledge of material parameters. We assess the contribution of electrons vs phonons to heat transport (see Figure 1c) using the microscopic charge conductivity obtained through all-optical electronic transport measurements.^32^ Our results show that—unlike in a typical metal—phonons play an important role in carrying in-plane heat in Ti_3_C_2_T_*x*_ MXene flakes.

**Figure 1:**
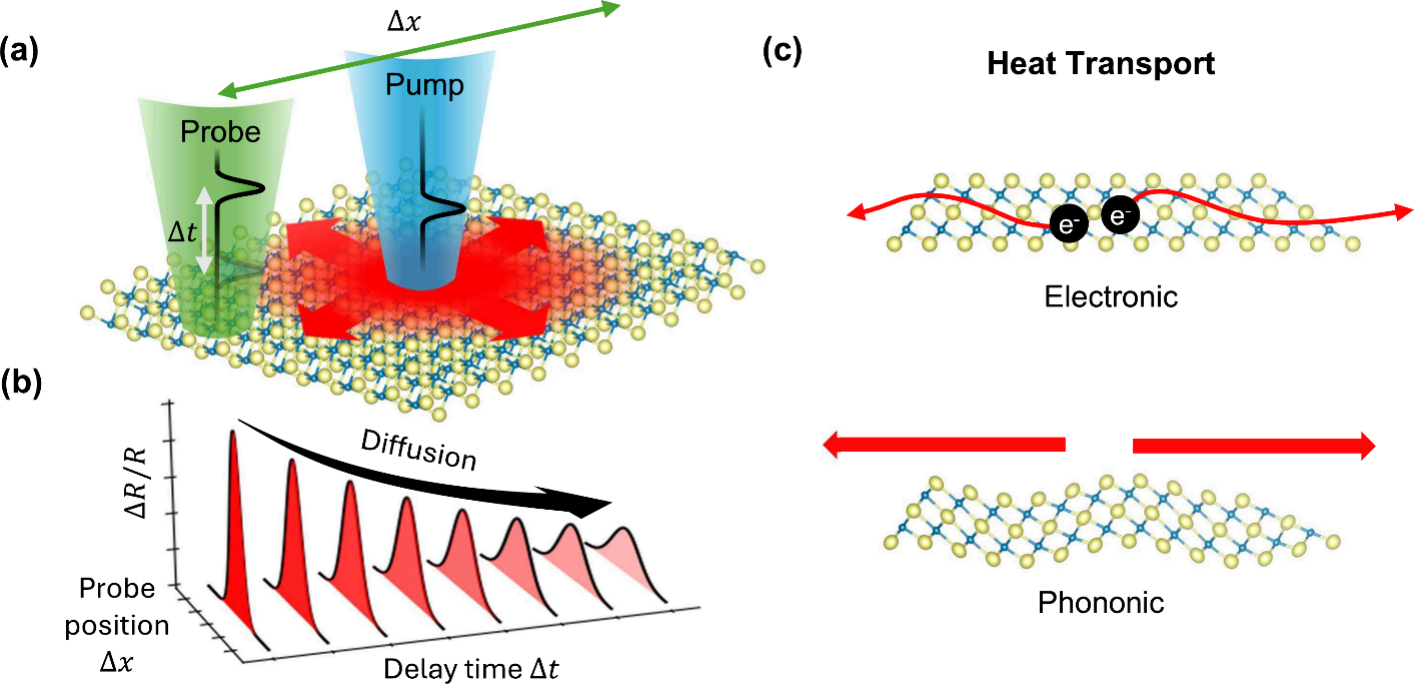
Spatiotemporal microscopy of heat transport by phonons and electrons. **a.** Schematic illustration of the principle of spatiotemporal microscopy to study in-plane heat transport. A pump pulse (blue) heats the central part of single-flake MXene. The heat (red) diffuses outward and a pump-induced reflectivity change *ΔR*/*R* is measured by the probe pulse (green) due to the temperature-dependent reflectivity around the plasmon resonance. The probe is offset relative to the pump in space and time by *Δx* and *Δt*, respectively. Spatiotemporal profiles are collected by scanning the probe in space and time. **b.** Evolution of the heat profile measured by spatiotemporal microscopy. Initially the profile matches that of the Gaussian-shaped pump pulse. With increasing *Δt*, diffusion causes this to spread out. **c.** Illustration of two different carriers of heat: electrons (top) and phonons (bottom).

The MXene flakes were synthesized by wet-chemical etching from the Ti_3_AlC_2_ MAX phase (as described in refs 26 and 33) and deposited onto Si/SiO_2_ substrates, starting from extremely dilute dispersions (0.01 to 0.001 mg/mL). From this, we obtained isolated MXenes flakes, allowing us to measure their intrinsic in-plane heat transport properties. We chose four specific flakes for our transport measurements, labeled flakes A, B, C, and D, which were chosen based on their size and uniformity. Atomic force microscopy (AFM) measurements showed that these flakes have thicknesses from 30 to 70 nm, while optical images showed lateral sizes varying between 5 and 20 μm (see Figures 2a,b and Supplementary Figure 6). We note that thermoreflectance measurements^26^ and scanning thermal microscopy measurements^19^ on identical Ti_3_C_2_T_*x*_ flakes showed highly inefficient out-of-plane heat transport to the substrate. Performing pump–probe measurements with varying laser repetition rate, similar to ref 32, allows us to estimate the out-of-plane heat flux, which is indeed low: 3.2–5.4 MW m^–2^ K^–1^ (see Supplementary Note 5). Therefore, it was not necessary to suspend the flakes, as was done in previous spatiotemporal heat transport measurements of 2D materials.^29^ For further details on the sample preparation and characterization, see Supplementary Notes 1 and 2.

**Figure 2:**
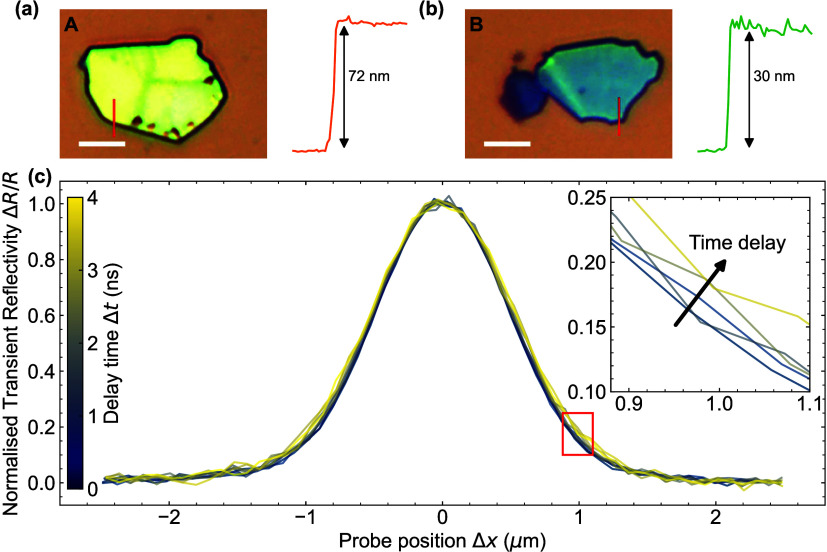
Spatiotemporal measurements of in-plane heat transport. **a**,**b.** Optical images together with AFM line traces for flake A (**a**) and flake B (**b**). The scale bars are 5 μm. **c.** Spatial transient reflectivity profiles *ΔR*/*R* of flake D, as a function of spatial pump–probe offset *Δx*, for five different time delays *Δt* between 0 and 4 ns (see color bar). These profiles correspond to spatial temperature profiles. The broadening of the profiles with time delay—due to heat transport—is on the order of tens of nanometers, as visible in the inset on the right.

We used spatiotemporal differential reflection microscopy^27–30^ to study heat transport. In this technique, illustrated in Figure 1a, a focused pump pulse with a duration of 50 ps and a wavelength of 590 nm locally photoexcites the center of a MXene flake. We then measure the pump-induced reflectivity change $\frac{\Delta R}{R}$ using a time-delayed probe pulse with a duration of 30 ps and a wavelength of ∼750 nm. At this wavelength, the reflectivity is governed by a temperature-dependent plasmon resonance^34–36^ of Ti_3_C_2_T_*x*_, and we operate in the regime where the signal scales linearly with pump and probe fluences (see Figures 3 and 4 of the Supporting Information). Therefore, scanning the probe beam over the pump spot, we obtain transient reflectivity profiles, which give the spatial distribution of the temperature change induced by the pump. As illustrated in Figure 1b, when the heat diffuses from the initial pump spot, this results in a broadening of the temperature—and thus transient reflectivity—profile. Measuring these profiles for different delay times between the pump and probe pulses enables us to follow in-plane heat diffusion. Due to inefficient out-of-plane thermal transport, the heat can stay in MXene flakes for more than 100 ns,^32^ which is longer than the inverse repetition rate of our laser (13 ns). To avoid heat accumulating in the flake, we therefore reduced the repetition rate of the laser by a factor 6 to 10, depending on the flake thickness, using a pulse picker as also discussed in Supporting Information Note 5. For more details on the measurement setup, see Supporting Information Note 2.

Figure 2c shows a typical set of normalized spatial profiles of the transient reflectivity at a few different delay times. We observe that the profiles broaden by ∼50 nm over 4 ns. We are able to resolve this small broadening because, as explained in detail in Supporting Information Note 2.1, our spatial resolution is not limited by the size of the pump and probe, but how stable their size is, alongside how precisely we can position the probe. Through the use of a high-precision scanning mirror, we calculate this to be around ∼7 nm for the probe position at the sample plane. To stabilize the pump and probe widths, we developed an autofocusing procedure which, as described in Supporting Information Note 2.3, provides a beam-width stability of <10 nm over >10 h.

To quantify the broadening of the transient reflectivity profiles, we plot the change in squared width of the profiles σ^2^–σ_0_^2^ as a function of delay time for four different flakes (see Figure 3a and Figure 7 of the Supporting Information for the complete data set). Here, σ is the Gaussian width of the profile at a certain delay time and σ_0_ is the width at time-zero, when the pump and probe pulses overlap in time. The slope of the squared width vs time provides the diffusivity *D*, as the solution of the diffusion equation for an initial Gaussian profile is σ^2^(*Δt*) = σ^2^(0) + 2*DΔt*, where we use the full time delay range between 30 ps (our time resolution) and 4 ns. Repeating this process for the four different flakes gives an average of *D* = 0.050 ± 0.002 cm^2^ s^–1^, resulting in a diffusion length of *L*_D_ = 140 nm over 4 ns. This diffusivity is significantly smaller than the thermal diffusivity of few-layer transition metal dichalcogenides (*D* > 0.15 cm^2^ s^–1^),^29,30^ silicon (0.6 cm^2^ s^–1^),^37^ and gold (1 cm^2^ s^–1^).^38^ This suggests relatively slow in-plane heat transport. We convert the thermal diffusivity to the thermal conductivity κ using κ = *C*_V_ *D*. Here, *C*_V_ is the volumetric heat capacity, for which values have been reported between *C*_V_ = 2.69 × 10 ^6^ J m^–3^ K^–1^ ^26^ and *C*_V_ = 3.2 × 10^6^ J m^–3^ K^–1^;^39^ see Supplementary Note 10. This gives an in-plane thermal conductivity of κ = 13–16 W m^–1^ K^–1^. Figure 3b shows the obtained thermal conductivities for the four flakes using the *C*_V_ from ref 26, giving an average in-plane thermal conductivity of κ = 13.5 ± 0.6 W m^–1^ K^–1^.

**Figure 3:**
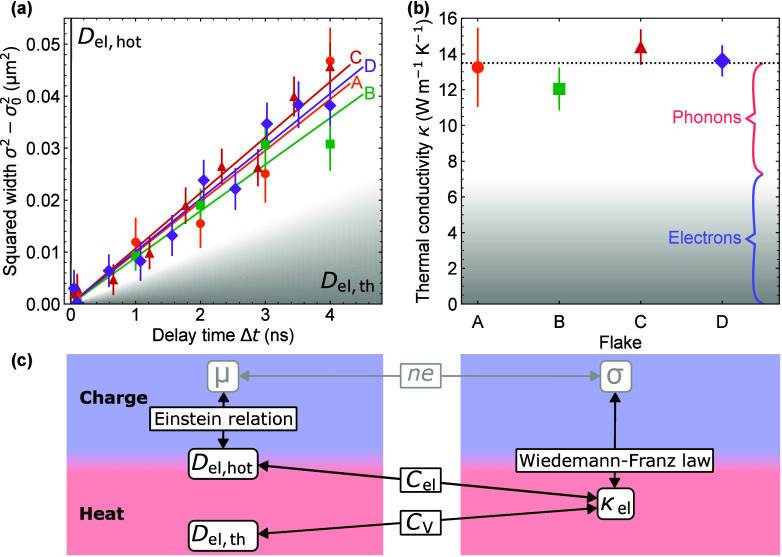
In-plane thermal diffusivity and conductivity. **a.** The change in squared width σ^2^–σ_0_^2^, obtained by describing the spatial profiles in Figure 2c and Figure 7 of the Supporting Information by a Gaussian function with standard width σ, as a function of time delay *Δt* for four different flakes. The slope is directly proportional to the thermal diffusivity *D*. The expected evolution of the squared width for diffusion of hot electrons *D*_el,hot_, before thermalization between electrons and phonons has occurred, is almost parallel to the vertical axis. The shaded area represents the expected evolution of the squared width for purely electronic heat transport after electrons and phonons have thermalized *D*_el,th_, for a range of different conductivities. The upper-bound is based on the microscopic charge conductivity obtained by terahertz transport measurements from ref 32, the application of Wiedemann–Franz law, and division by the heat capacity *C*_V_ from ref 26. **b.** The heat conductivity calculated by multiplying the obtained diffusivities for the different flakes, by the heat capacity *C*_V_ with the weighted average (dotted line). The braces show the contributions from electrons and from phonons to the heat transport, indicating the importance of phonons for in-plane heat transport. **c.** Schematic showing how microscopic and macroscopic properties describing heat and charge transport are connected. In particular, the electrical conductivity and mobility are related via the carrier density *n*, electron charge *e*, and the charge mobility μ, while the diffusivity of hot electrons *D*_el,hot_ and of thermalized electrons *D*_el,th_ are related to the electronic contribution to the thermal conductivity via the heat capacity.

Before we discuss the relative contributions of electrons and phonons to in-plane heat transport in our MXene flakes, we first describe how ultrafast heat diffusion occurs in typical metals, using gold as an example.^28,38^ Initially, photoexcitation introduces energy purely into the electronic system. Through strong electron–electron interactions, within tens of fs, thermal equilibrium within the electron system is established, well before equilibrium with the lattice. As illustrated in Figure 3c, the diffusivity of these hot electrons *D*_hot,el_ is related to the electronic thermal conductivity κ_el_ and the electronic heat capacity *C*_el_ as $D_{\mathrm{hot},\mathrm{el}}=\frac{\kappa_{\mathrm{el}}}{C_{\mathrm{el}}}$ . The thermal conductivity κ_el_ is related to the charge conductivity σ_el_ via the Wiedemann–Franz law:^40,41^ $\kappa_{\mathrm{el}}=\frac{\pi^{2}}{3}\frac{k_{B}^{2}}{e^{2}}T\sigma_{\mathrm{el}}$ , where *k*_B_ is Boltzmann’s constant, *e* is electron charge, and *T* is temperature. The hot electron diffusivity in this first regime also follows directly from the charge mobility, as we illustrate in Figure 3c and show in Supporting Information Note 9. In gold, the hot-carrier diffusivity in this regime is around 100 cm^2^ s^–1^.^38^

After some time, the electron and phonon systems have fully equilibrated, and a second regime begins. At this point, the heat is shared between the electron and phonon systems. Thus, as shown in Supporting Information Note 8, the diffusivity is governed by both the electronic and phononic conductivities and heat capacities: $D_{\mathrm{th}}=\frac{\kappa_{\mathrm{el}}+\kappa_{\mathrm{ph}}}{C_{\mathrm{el}}+C_{\mathrm{ph}}}$ , where κ_ph_ and *C*_ph_ are the thermal conductivity and heat capacity of the phonons, respectively. As the heat capacity of phonons is much larger than that of electrons, *C*_ph_ ≫*C*_el_, and the electrical conductivity of conventional metals is high, κ_el_ ≫κ_ph_; this simplifies to $D_{\mathrm{th}}\approx \frac{\kappa_{\mathrm{el}}}{C_{\mathrm{ph}}}$ . In gold, the diffusivity observed in this regime is around 1 cm^2^ s^–1^,^38^ which, using its phonon heat capacity, corresponds to the thermal conductivity of gold, which is around 250 W m^–1^ K^–1^.

Returning to our Ti_3_C_2_T_*x*_ MXene flakes, we note that in the first regime, where electrons are not thermalized with the lattice, we expect a diffusivity of the hot electrons of tens of cm^2^ s^–1^. This value is based on the charge mobility and Fermi level of similar flakes, as obtained using terahertz spectroscopy measurements from ref 32 (see Supporting Information Note 9). Since we observe a much smaller diffusivity in our experiment, we conclude that our measurements correspond to the second regime, where electrons and phonons are in equilibrium. This is also consistent with earlier pump–probe measurements that suggested (sub-)­picosecond electron–phonon equilibration,^42^ while our time resolution is several tens of picoseconds.

Having established that the obtained diffusivity corresponds to the thermal conduction of equilibrated electrons and phonons, we assess their separate contributions. For the electronic contribution, we use the Wiedemann–Franz law to determine the electronic contribution to the thermal conductivity. For this, we consider a range of charge conductivities of σ_el_ = 10^4^–10^6^ Sm^–1^. The upper limit is given by the number obtained through all-optical microscopic transport measurements with terahertz pulses^32^ and represents a microscopic conductivity that is unaffected by grain boundaries, etc. The lower limit corresponds to values that were obtained using electrical transport measurements in other studies, which can be up to 2 orders of magnitude lower.^43^ In particular, studies found conductivities of 9 × 10^–5^ S m^–1^,^44^ 4 × 10^5^ S m^–1^,^45^ 5 × 10^5^ S m^–1^,^16^ and 6.5 × 10^5^ S m^–1^.^46^ Using this range of values, the electrical contribution to the thermal conductivity will be at most κ_el_ = 7.3 W m^–1^ K^–1^. As illustrated in Figure 3b, this value is significantly lower than our measured thermal conductivity, which is 13–16 W m^–1^ K^–1^. This indicates that phonons must play an important role in the in-plane thermal conductivity of Ti_3_C_2_T_*x*_ MXene. In this analysis, we assumed purely diffusive transport by using a Lorenz number of 1. In ref 19 a violation of the Wiedemann–Franz law was reported, with a Lorenz number of around 0.25 for monolayers of Ti_3_C_2_T_*x*_. If similar nondiffusive transport would occur in the thicker flakes that we studied here, the contributions of electrons to heat transport would be four times smaller, meaning that phonons would be an even more dominant carrier of heat.

In typical metals, the thermal conductivity is dominated by electrons, while we found a significant role of phonons for our metallic Ti_3_C_2_T_*x*_ MXene flakes. We illustrate this deviation from the typical metallic behavior in Figure 4a, which shows the thermal conductivity κ of several materials as a function of charge conductivity σ_el_. Here, we see that the elemental metals nicely follow the Wiedemann–Franz law. Some alloys, two-dimensional metallic compounds, and semiconductors divert from this line, exhibiting a larger thermal conductivity due to phononic contributions. Interestingly, the metallic MXene Ti_3_C_2_T_*x*_ has a unique combination of relatively large charge conductivity with a relatively low thermal conductivity. Making use of the carrier density obtained by THz measurements,^32^ in Figure 4b, we compare thermal conductivities as a function of carrier density. Here we find that the reason for the relatively low electronic contribution to the thermal conductivity in Ti_3_C_2_T_*x*_ is the low carrier density of *n* = 1.2 × 10 ^21^ cm^–3^, compared to, for example, 5.9 × 10 ^22^ cm^–3^ for gold (see Figure 4b). This may relate to predictions of a relatively low density of states—for a metal—around the Fermi level.^54^ Figure 4c shows that Ti_3_C_2_T_*x*_ MXene furthermore has a unique combination of relatively large charge mobility and relatively low thermal conductivity. This is different from the case of alloys, where the mobility is reduced due to increased scattering. A better understanding of this combination of properties could be sought through further studies in which, for example, the carrier concentration or mobility of Ti_3_C_2_T_*x*_ is tuned either chemically or externally via gating. Regardless, this comparison shows that Ti_3_C_2_T_*x*_ has an unconventional combination of charge transport and heat transport properties, which is useful for thermal management applications.

**Figure 4:**
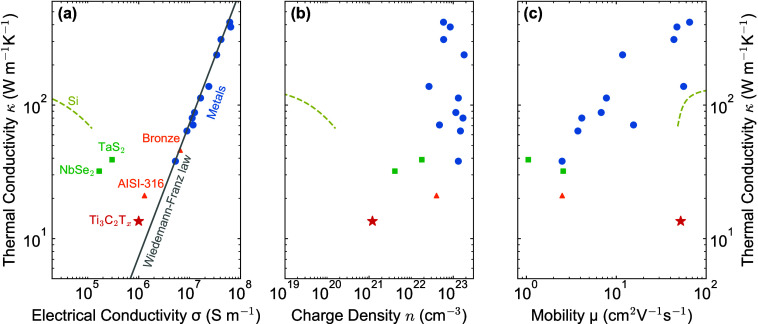
Comparison of heat and charge transport properties in different materials. The thermal conductivity as a function of electrical conductivity (a), electron density (b), and electrical mobility (c) of elemental metals (blue ●), from refs 47 and 48, alloys (orange ▲), from ref 49, metallic compounds (green ■), from refs 50 and 51, B-doped silicon with different concentrations (dashed line), from refs 52 and 53, and Ti_3_C_2_T_*x*_ (red ★) (this work and ref 32).

In conclusion, we have measured the thermal diffusivity of single multilayer flakes of Ti_3_C_2_T_*x*_ MXene using spatiotemporal microscopy. This technique enabled us to obtain the intrinsic thermal diffusivity without any prior knowledge of material parameters. By measuring single flakes, we eliminated the effects of flake size and interflake thermal resistance. We obtained an in-plane thermal diffusivity of *D* = 0.050 ± 0.002 cm^2^ s^–1^, corresponding to a thermal conductivity of κ = 13–16 W m^–1^ K^–1^. This is comparable to the thermal conductivity measured for other thick flakes,^23^ but much higher than for monolayer flakes, where a value of 0.78 W m^–1^ K^–1^ was found. That value is even lower than the value expected based on electron-dominated heat transport following the Wiedemann–Franz law, suggesting a negligible contribution of phonons and nondiffusive electronic heat transport.^19^ This suggests a significant dependence of the in-plane thermal conductivity on MXene flake thickness and in particular a strongly decreasing contribution of phonons to heat transport upon approaching the monolayer thickness limit. This requires further research into the thickness dependence of the in-plane thermal conductivity, since differences in termination group composition could also play a role. We compared the upper limit for the electronic contribution to the thermal conductivity, obtained using the electrical conductivity from THz measurements,^32^ to the phononic contribution and concluded that phonons play an important role. We then compared the electrical and thermal transport properties to that of other materials and found that the low electronic contribution to the thermal conductivity is not caused by a low mobility, as is the case in alloys and other 2D metallic compounds, but by a relatively low carrier density.

With these in-plane thermal transport measurements, we have shed light on the nature of heat conduction in the Ti_3_C_2_T_*x*_ MXene. Our results show the importance of phonons for in-plane thermal transport, which should be taken into account when designing devices based on these materials. Moreover, the low carrier density could open up ways of tuning the thermal conductivity by changing the carrier density through, e.g., gating or intercalation. This could, for example, be used to create a smart thermal material for active thermal management in electronic devices, batteries, and smart textiles.^55,56^

## Supplementary Material



## Notes

Additional details on the experimental methods (Notes 1–7) and theory (Notes 8 and 9), including 7 figures with additional experimental results and descriptions (PDF)
