# Supplementary material for: Phonon-Dominated In-Plane Thermal Conductivity of Single-Flake Metallic Ti3C2T x MXene
Source: Nano Lett. 2026 Jul 30;26(31):10181–7. doi: 10.1021/acs.nanolett.6c01850 (PMC13475044; doi:10.1021/acs.nanolett.6c01850)
Supplement: Supplementary file 1 [file nl6c01850_si_001.pdf]

Supporting Information for Phonon-dominated in-plane thermal conductivity of single-flake metallic $Ti_3C_2T_x$ MXene

Max C. van Hemert[1], Hugh Ramsden[1], Stefano Ippolito[2], Sebin Varghese[1], Bohai Liu[1], Elvira Tornero Gasca[3], Miguel Muñoz Rojo[3,4], Hai I. Wang[5,6], Yury Gogotsi[2], and Klaas-Jan Tielrooij[1,7]

[1]Department of Applied Physics and Science Education, Eindhoven University of Technology, Den Dolech 2, Eindhoven, 5612 AZ, The Netherlands
[2]A.J. Drexel Nanomaterials Institute and Department of Materials Science and Engineering, Drexel University, Philadelphia, PA 19104, USA
[3]Energy2Devices, Instituto de Ciencia de Materiales de Madrid (ICMM), Calle Sor Juana Inés de la Cruz 3, Madrid, 28049, Spain
[4]Department of Thermal and Fluid Engineering, Faculty of Engineering Technology, University of Twente, Enschede, 7500 AE, The Netherlands
[5]Max Planck Institute for Polymer Research, Ackermannweg 10, Mainz, 55128, Germany
[6]Debye Institute for Nanomaterials Science, Utrecht University, Princetonplein 1, Utrecht, 3584, The Netherlands
[7]Catalan Institute of Nanoscience and Nanotechnology (ICN2), CSIC and BIST, Campus UAB, Bellaterra, Barcelona, 08193, Spain

*Email: k.j.tielrooij@tue.nl

This file includes:

- 1: MXene Synthesis and Sample Preparation
- 2: Spatiotemporal Microscopy
- 3: Transient Reflectivity Spectrum
- 4: Linearity with Laser Power
- 5: Repetition Rate Dependence and Out-of-plane Heat Transport
- 6: Transient Reflectivity Maps
- 7: Spatiotemporal Measurements
- 8: Two-temperature Model
- 9: Electronic thermal diffusivity and conductivity

1 MXene Synthesis and Sample Preparation

The samples have been prepared in the same manner as described by Tornero Gasca *et al.*[7] In short, $Ti_3C_2T_x$ MXene flakes have been obtained by wet-chemical etching of the Ti_3AlC_2 MAX phase, supplied by Carbon-Ukraine, using the protocol of Downes *et al.*[11] This process yields fully delaminated MXene flakes without residual MAX phase, which have been extensively characterised before.[15] The delaminated flakes were dispersed in water at a concentration of 0.01 to 0.001 mg mL^{-1} and deposited on $Si\backslash SiO_2$ substrates by dip coating.

2 Spatiotemporal Microscopy

We measured the thermal diffusivity using spatiotemporal microscopy.[13,14] A schematic of the setup is illustrated in Fig. 1. An NKT Photonics SuperK Fianium Fiu-6 supercontinuum laser is used to generate white light pulses, from which a pump (590 nm) and probe (720 or 780 nm) wavelength are selected using an NKT Photonics SuperK Select acousto-optical tunable filter (AOTF). These are separated onto different paths using a Thorlabs DMLP650 dichroic mirror. The pump beam is modulated at a frequency of 4.37 kHz using a mechanical chopper, and next sent over a Standa 8MT-195X-1040 delay line. The probe beam is reflected by an Optics in Motion OIM 101 scanning mirror, which, in combination with a 4f-lens system, allows us to scan the probe beam over the pump spot on the sample. The beams are combined by another dichroic mirror and focused on the sample to a spot size of 0.55 µm to 0.77 µm (FWHM) using an OptoSigma PAL-50-NIR-HR-LC00 objective which also collects the reflected light. The reflected light is separated from the incident light using a beam splitter, and the reflected pump is filtered out using a Thorlabs FELH0650 long-pass filter. The reflected probe light is focused onto a Thorlabs DET10A2 photodiode. The signal is collected and demodulated using a Zurich Instruments MFLI lock-in amplifier to extract the transient reflectivity $\frac{\Delta R}{R} = \frac{R_{\text{pump on}} - R_{\text{pump off}}}{R_{\text{pump off}}}$.

The transient reflectivity profiles are measured by scanning the position of the probe beam with respect to the pump beam using the scanning mirror, which is repeated at different time delays. We verified a linear dependence of the signal with pump power, such that the spatial profiles properly represent temperature profiles (see Note 4). We determined the widths of the temperature profiles by describing the spatial profile with a Gaussian function:

$$\frac{\Delta R}{R}(\Delta x, \Delta t) = Ae^{-\frac{\Delta x^2}{2\sigma^2(\Delta t)}}, \tag{1}$$

where $\sigma(\Delta t)$ is the width of the heat profile at time delay Δt, Δx is the spatial offset between the pump and probe pulse, and A is the amplitude of the relative reflectivity change. The resulting squared widths are plotted as a function of delay time in Fig. 2c, and provide the obtained diffusivities. Since we use tightly focused light and measure only a very small broadening of the temperature profile, a uniform area of only $\sim 4\,\mu$m on a flake is needed, as is visible in the transient reflectivity maps in Supplementary Fig. 6.

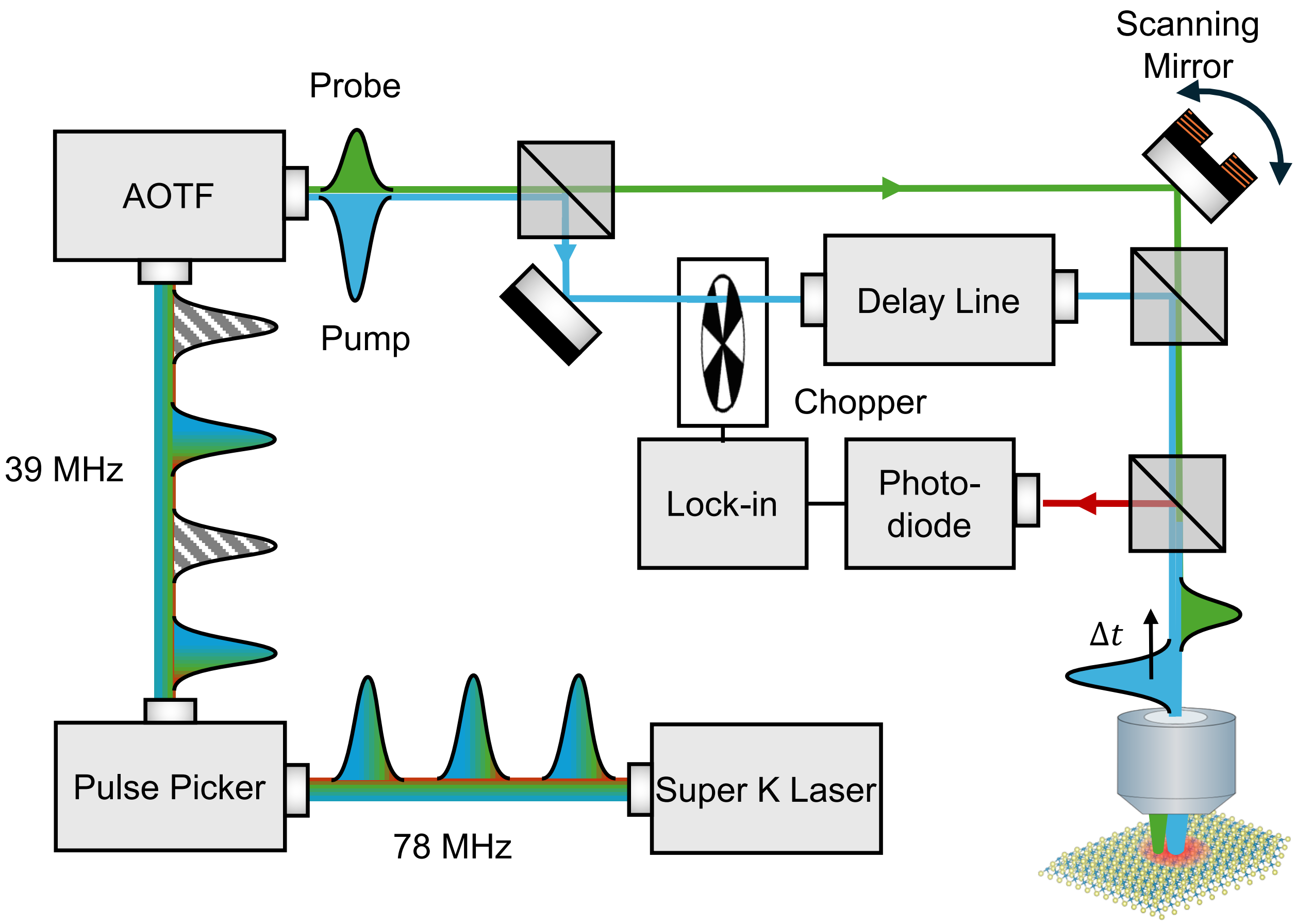

Figure 1: **Schematic diagram of the spatiotemporal setup.** Pulsed white light is generated by a supercontinuum laser. The repetition rate is reduced by a pulse picker. Pump and probe wavelengths are selected using an acousto-optical tunable filter (AOTF). These are separated and later overlapped using dichroic mirrors. The pump is modulated by a chopper and travels along a delay line. The probe is steered by a scanning mirror. Both beams are focused using an objective on the sample. The reflection of the probe is focused on a photodiode, which is connected to a lock-in amplifier synchronised to the chopper.

2.1 Spatial Resolution

Unlike typical optical spectroscopies, spatiotemporal microscopy is not diffraction limited in its spatial resolution. To understand this, we start by expressing the pump-induced temperature increase as a function of offset from the beam centre, Δx, and delay after pump pulse arrival, Δt,

$$\Delta T(\Delta x, \Delta t) = A_T(t) e^{-\frac{\Delta x^2}{2(\sigma_{\text{pump}}^2 + 2D\Delta t)}}, \tag{2}$$

where $A_T(t)$ is the time-dependent amplitude change, σ_{pump} is the pump width, and as described in the main text, D is the thermal diffusivity.

As the probe scans over the sample, the overall $\Delta R/R$ profile observed is the convolution of the probe profile with the temperature profile. This means the resulting profile can be expressed as

$$\frac{\Delta R}{R}(\Delta x, \Delta t) = A_R(t) e^{-\frac{\Delta x^2}{2\left(\sigma_{\text{pump}}^2 + \sigma_{\text{probe}}^2 + 2D\Delta t\right)}}, \tag{3}$$

where $A_R(t)$ is a different time dependent amplitude, with $A_R(t) \propto A_T(t)$, and σ_{probe} is the width of the probe. From this we see the measured $\Delta R/R$ profile width, σ, can be expressed as

$$\sigma(\Delta t)^2 = \sigma_{\text{pump}}^2 + \sigma_{\text{probe}}^2 + 2D\Delta t, \tag{4}$$

thus the diffusivty can be extracted via $\sigma^2 - \sigma_0^2 = 2D\Delta t$ (where σ_0 is the width at $\Delta t = 0$), from which it can be seen that both the width of the pump and probe do not play a role. Instead, the ability to resolve the diffusive contribution, $2D\Delta t$, depends on minimizing uncertainties in Δx, σ_{pump} and σ_{probe}, which we achieve through the use of a high precision scanning mirror and autofocusing procedures, outlined below.

2.2 Spatial Scanning Resolution

The probe beam is steered using a scanning mirror that is imaged onto the back aperture of the objective (focal length f_{obj}) through a 4-f relay composed of two lenses with focal lengths f_1 and f_2. In a conjugate configuration, angular deflections of the mirror are mapped into lateral displacements at the sample plane, while preserving the focal plane. The relation between the lateral scan range R_{pr} and the mirror scan angle Φ_{pr} is[1]

$$R_{\text{pr}} = \Phi_{\text{pr}} \frac{f_1 f_{\text{obj}}}{f_2}. \tag{5}$$

For the optical layout used in this work ($f_1 = 250$ mm, $f_2 = 300$ mm, $f_{\text{obj}} = 4$ mm), a full mirror angle of 3° (≈ 52 mrad) produces a scan range of approximately 170 μm at the sample plane. Given the mirror's angular resolution of about 2 μrad, the corresponding spatial resolution of the scanning system is around 7 nm.

2.3 Autofocus Procedure

As described further in Supplementary Note 5, a reduced repetition rate was required to eliminate heat accumulation effects. This resulted in a lower signal, thus requiring long acquisitions, over several hours, to achieve sufficient signal-to-noise ratios. Therefore, to resolve nm changes in the transient reflectivity profile, it was essential to maintain optimal focus throughout the measurement. This was achieved through the use of an automated focusing procedure. For this, we used the coordinate system illustrated in Fig. 2a. As illustrated in Figs. 2b and c, the strongest response $\Delta R/R$ is measured for the best pump-probe overlap, and at optimal focus. Therefore, to focus the system, the gradient of $\Delta R/R$ with respect to x and y was measured for a given pump-probe overlap, then, a step forward was taken proportional to this change. However, as indicated by the dashed arrow in Figure 2b, close to perfect overlap, $\Delta R/R$ is very sensitive to probe position, which could result in over-stepping the optimum. To avoid this, a dampening factor was added that attenuated the step size, such that it was always less than the beam width. Initially, the pump-probe overlap is optimized in the x-y plane, then the focus in z is optimized, shown in Fig. 2c. To avoid the accumulation of hysteresis in movement along each axis, when measuring gradients, the small step was alternately taken in positive and negative directions.

To verify the stability of the focus, we performed successive knife-edge measurements[2] over 18 hours, autofocusing before each knife-edge. As illustrated in Fig. 2d, these knife-edge measurements were very stable. In Fig. 2e, we plot the distribution of probe widths calculated by fitting the knife-edge responses with a Gaussian profile. These show the majority of σ values remaining within $\pm$ 10 nm. The source code for this focusing procedure has been made available online.[3]

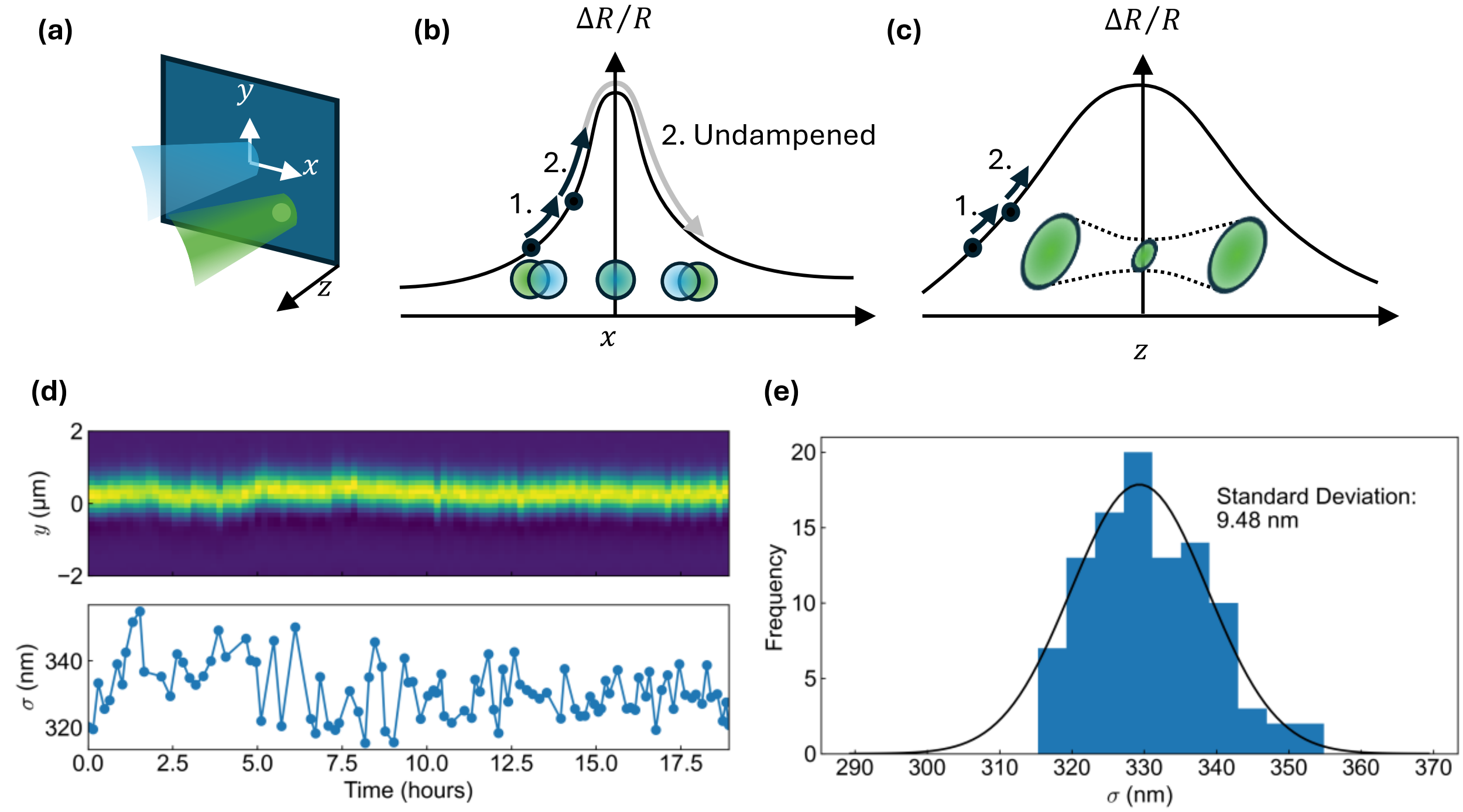

Figure 2: **Description of autofocusing procedure. a** Coordinate system used. x and y indicate the position of the probe, shown green, relative to the pump, shown blue. These are modified using the scanning mirror, while z is changed by moving the sample stage away from or towards the objective. **b** Illustration of dependence of $\Delta R/R$ on pump-probe overlap x. The same relationship also applies to y. **c** Dependence of $\Delta R/R$ on stage z position. **d** Stability of autofocused probe. Top, plot of successive knife-edge scans, using a procedure based on.[2] The colour-scale represents $\partial R/\partial y$. Bottom, plot of probe widths obtained from Gaussian fits of knife-edge scans. **e** Histogram of knife-edge probe widths in **d**. The normal distribution fit, shown black indicates a standard deviation of 9.48 nm.

3 Transient Reflectivity Spectrum

$Ti_3C_2T_x$ MXenes have a plasmon resonance at a wavelength of 720 nm.[4] Because the reflectivity around resonance is temperature dependent,[5] we use the change in reflectivity as a proxy for the change in temperature. An increase in temperature corresponds to a decrease in reflectivity. To maximize the change in reflectivity ΔR for the same pump-induced temperature change, we measure the transient reflectivity as a function of probe wavelength, shown in Fig. 3. We see that the maximum signal is indeed obtained for a probe wavelength around 750 nm, close to the plasmon wavelength.[6] From this figure, it can also be seen that the transient reflectivity signal from the substrate is much lower than the signal from the MXene flake around this wavelength. This indicates that in the spatiotemporal measurements the signal is dominated by the response of the MXene, especially because the substrate is covered by the flake.

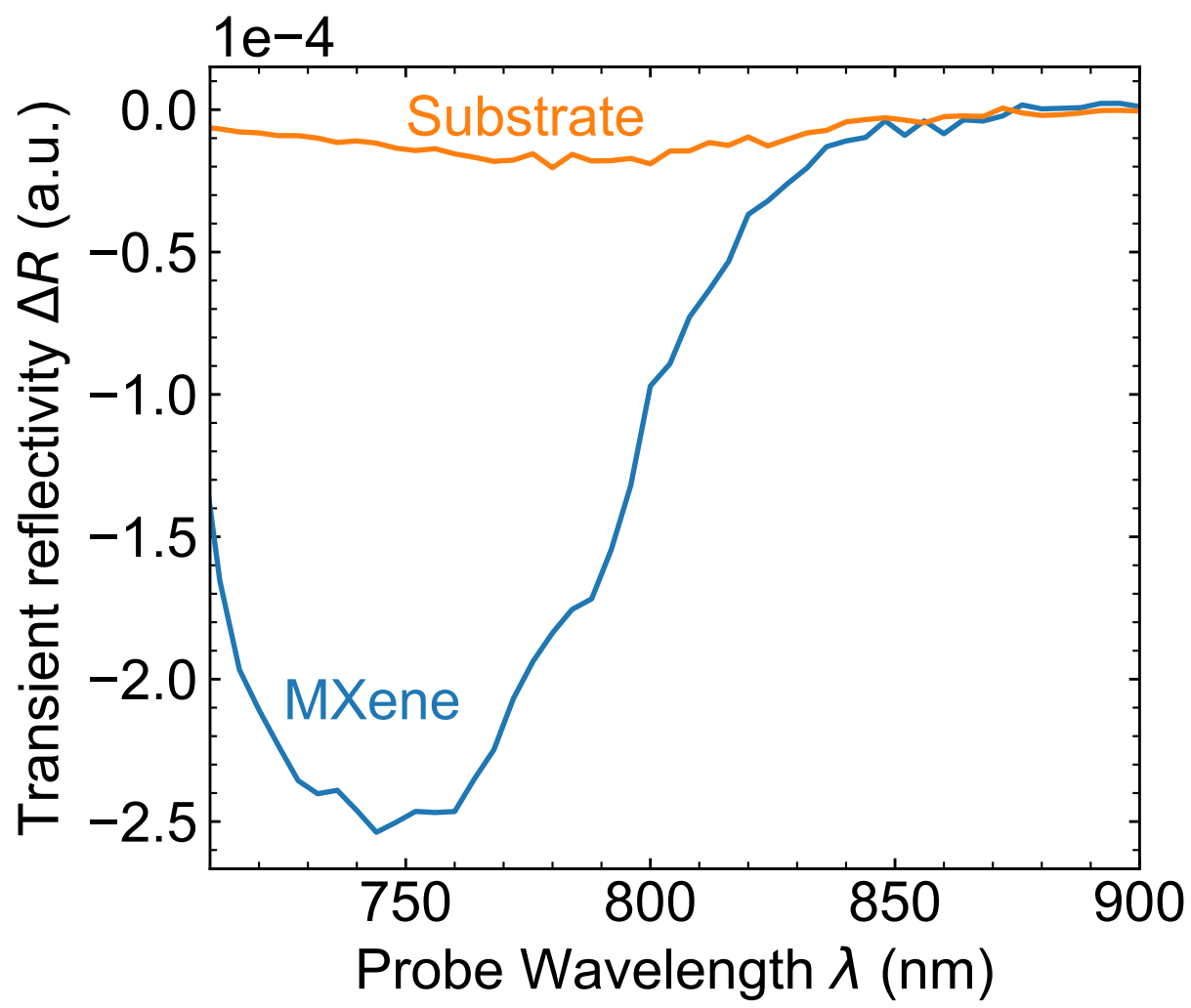

Figure 3: **Transient reflectivity spectrum** for the MXene flake (blue) and substrate (orange) as function of probe wavelength.

4 Linearity with Laser Power

Provided we operate in the linear-absorption regime, the pump-induced temperature change should be proportional to the excitation fluence P. For spatiotemporal measurements, we seek to operate in the regime where ΔR is proportional to the pump-induced temperature change ΔT. To ensure this is the case, in Figs. 4a and b, we plot ΔR with respect to both pump and probe fluences. Here we see that up to a pump fluence of 250 μJcm^{-2}, and a probe fluence up to 340 μJcm^{-2}, the response remains linear. Therefore to ensure interpretability of the spatiotemporal profiles obtained in this study, we selected 250 μJcm^{-2} and 180 μJcm^{-2} for the pump and probe fluences respectively. To further ensure non-linearities were not influencing the observed dynamics in Figs. 4c and d, we plot normalized time traces taken with a range of pump and probe fluences. Here we see that in all cases the same dynamics are observed. Taking a repetition rate of 78 MHz, it is noteworthy in these time traces that the heat has not completely decayed when the next pulse arrives after 13 ns. In Figs. 4a and b, we also demonstrate that this residual pre-time-zero signal is linear with the pump and probe fluence.

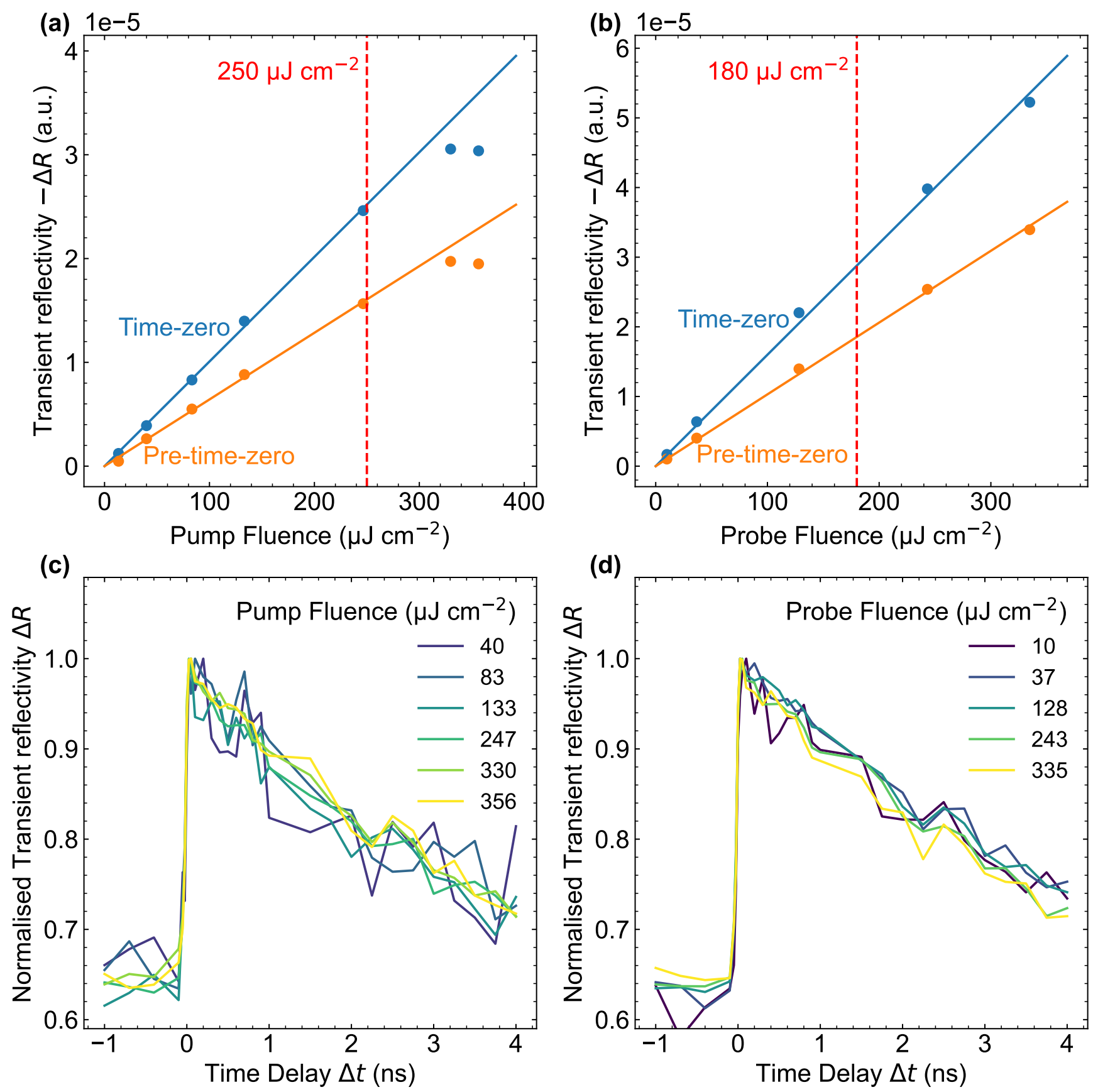

Figure 4: **Excitation fluence dependent dynamics**. **a,b** The amplitude of the transient reflection signal ΔR of flake A as function of pump and probe fluence, respectively, showing both the time-zero signal (/) and the accumulated pre-time-zero signal from previous pulses (/), measured at a repetition rate of 78 MHz for spatially overlapped pump and probe. Straight lines are drawn as a guide to the eye. The dashed vertical lines indicate the fluence used in the other measurements in this research. **(c,d)** The decay dynamics of the transient reflection signal ΔR for overlapped pump and probe, normalised to the maximum value, for multiple pump and probe fluences, respectively.

5 Repetition Rate Dependence and Out-of-plane Heat Transport

As visible in the time traces in Fig. 4, the temperature decays very slowly, which has also been discussed previously.[7,8] This could result in spatiotemporal profiles that cannot be described by a single Gaussian function due to residual heat from previous pump pulses. To eliminate this residual heat from earlier pulses, visible as pre-time-zero signal, we used a pulse picker to reduce the repetition rate f_{rep} and increase the time between the pulses. As shown in Fig. 5a, this reduces the residual pre-time zero signal. However, this also reduces the amplitude of the measured transient reflection, because there are fewer probe pulses. To correct for this effect, we normalize the transient reflection by the steady-state reflection R in Fig. 5b. This shows that the amplitude of the time-zero peak is constant for different repetition rates, while the residual signal is reduced. This is also visible in Fig. 5c, where the residual signal is reduced drastically, while the temperature change induced by the pump stays constant.

We use the decrease of the residual signal at lower repetition rates to determine the thermal dissipation time τ_{diss} by modelling the measured signal at different repetition rates. A probe pulse measures the accumulated temperature change by all previous pump pulses i, which have decayed for a time of $\frac{i}{f_{\mathrm{rep}}}$. This results in the following equation

$$\frac{\Delta R_{\mathrm{residual}}}{R} \propto \sum_{i=1}^{N_{\mathrm{pulses}}} \exp\left(-\frac{i}{f_{\mathrm{rep}}\tau_{\mathrm{diss}}}\right), \tag{6}$$

where $N_{\mathrm{pulses}} = \frac{f_{\mathrm{rep}}}{2f_{\mathrm{mod}}}$ is the number of pump pulses per modulation window. The factor two is to account for the time when the pump is blocked by the chopper. Applying this model to the repetition-rate-dependent time traces for flakes A and B, results in a dissipation time $\tau_{\mathrm{diss,A}} = 43 \pm 3\,\mathrm{ns}$ and $\tau_{\mathrm{diss,B}} = 26 \pm 3\,\mathrm{ns}$, as shown in Fig. 5c. We then obtain the out-of-plane heat flux G using

$$G = \frac{C_V d}{\tau_{\mathrm{diss}}}, \tag{7}$$

where C_V is the heat capacity and d the thickness of the flake. Using the range of heat capacities $C_V = 2.7 - 3.2 \times 10^6\,\mathrm{Jm^{-3}K^{-1}}$ and the flake thickness as measured using AFM gives the out-of-plane thermal conductance in the range of $3.2 - 5.4\,\mathrm{MWm^{-2}K^{-1}}$. This aligns well with the results of Ref.,[7] where flakes with a thickness between 30 and 70 nm were found to have a G of $2.5 - 5\,\mathrm{MWm^{-2}K^{-1}}$, limited by the out-of-plane thermal conductivity.

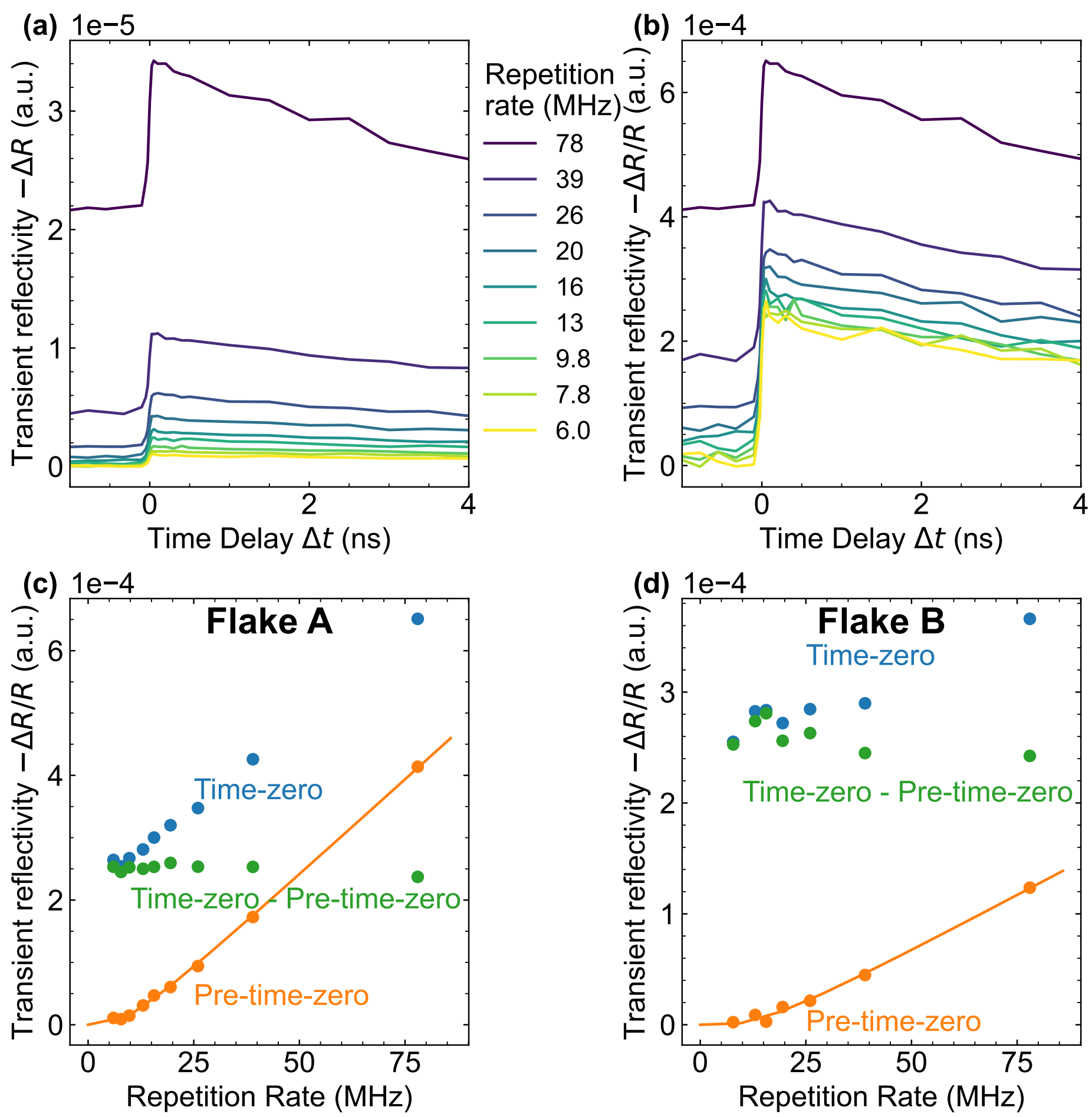

Figure 5: **Repetition-rate-dependent dynamics**. **a** Decay dynamics of the transient reflection signal ΔR on flake A for spatially overlapped pump and probe for multiple repetition rates. **b** Same as panel **a**, now normalised to the steady-state reflectivity R. **c,d** Amplitude of the transient reflection signal as a function of repetition rate, showing the time-zero amplitude (•), the pre-time-zero signal, accumulated from previous pulses (•), and the difference between these two (•), for flakes A and B, respectively. We use the residual heat model Eq. 6 to describe the pre-time-zero amplitude (/)

.

6 Transient Reflectivity Maps

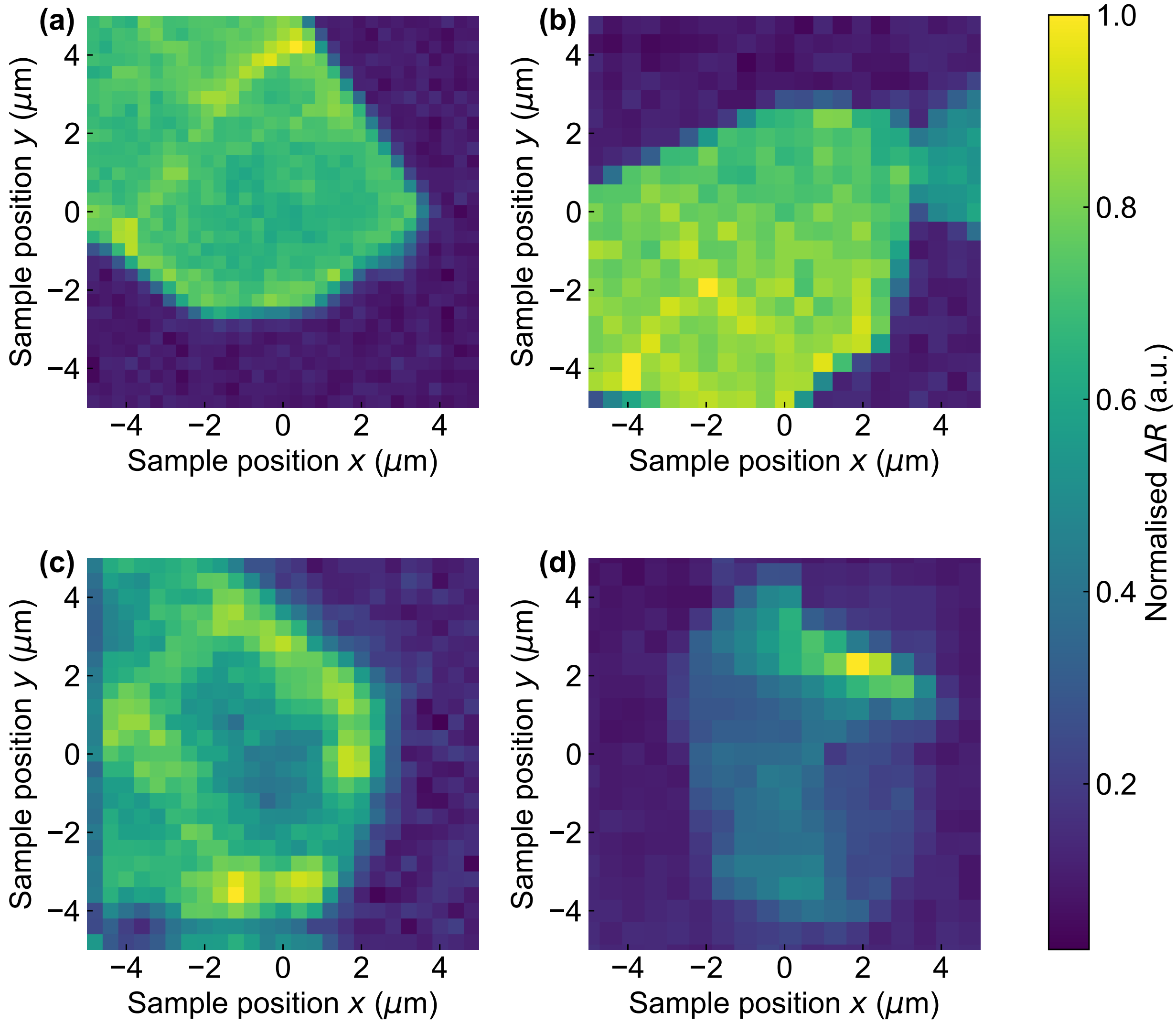

Figure 6: **Transient reflectivity maps.** Normalised $\Delta R/R$ for spatially and temporally overlapped pump and probe, as function of position around the region of interest, for flakes A-D in panel **a-d**, respectively.

7 Spatiotemporal Measurements

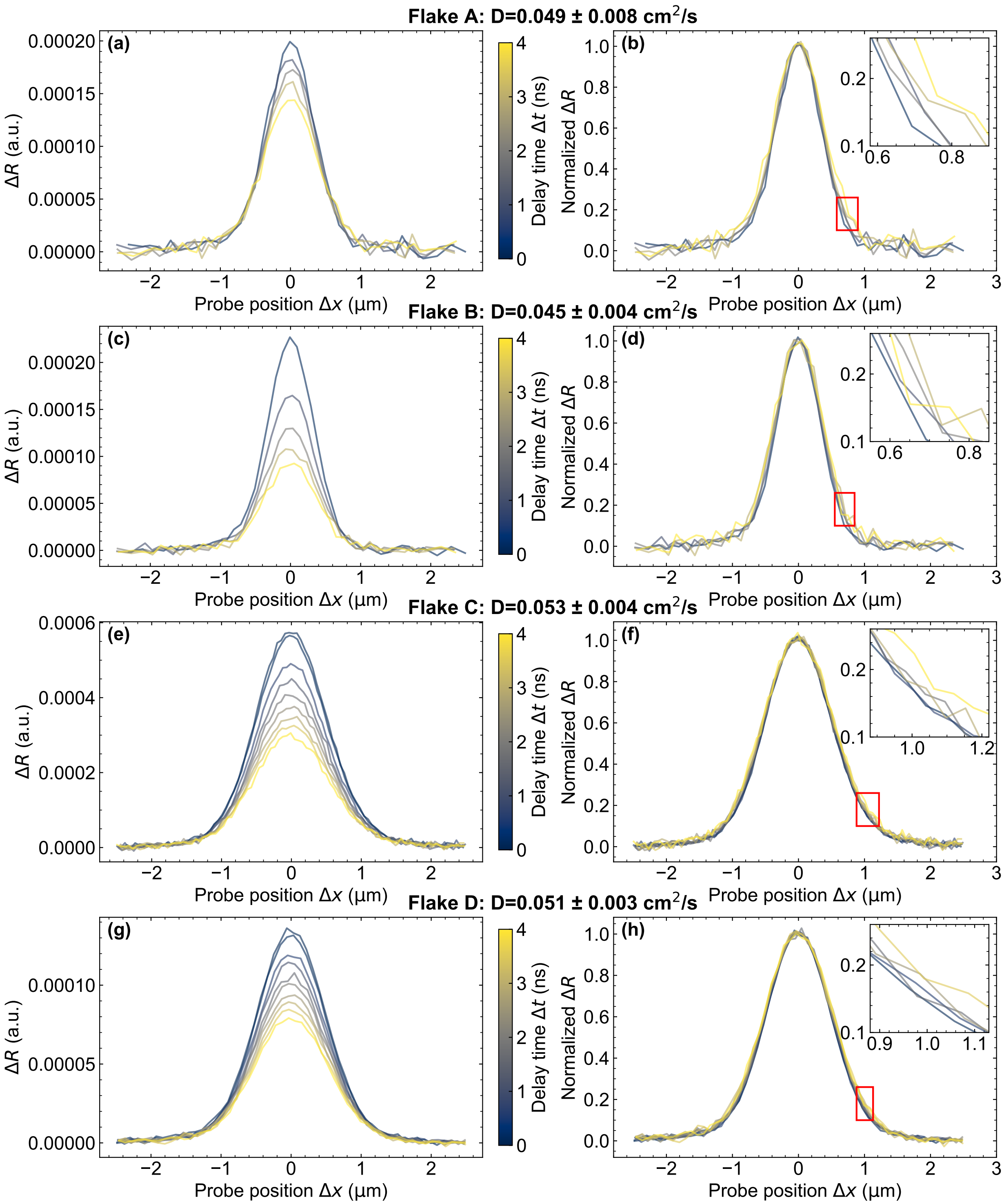

Figure 7: **Spatiotemporal profiles for all flakes.** Transient reflectivity ΔR (**a**, **c**, **e**, **g**) and normalised transient reflectivity (**b**, **d**, **f**, **h**) as function of probe position, at different time delays for the different flakes A (**a-b**), B (**c-d**), C (**e-f**) and D (**g-h**).

8 Two-temperature Model

When the pump pulse is absorbed by the sample, this excites some electrons. Through electron-electron thermalization, the energy is distributed among all electrons, but still unthermalized with the phonons. This results in a thermal distribution of "hot" electrons with a temperature T_{el} and heat capacity C_{el}. In metals like gold, this thermalization generally happens within tens of femtoseconds, faster than the thermalization with the phonons, which is governed by the electron–phonon coupling coefficient $G_{\mathrm{el-ph}}$.[6] The coupling between the electron and phonon subsystems and subsequent diffusion are described by a two-temperature model

$$C_{\mathrm{el}}\frac{\partial T_{\mathrm{el}}}{\partial t} = \kappa_{\mathrm{el}}\nabla^2\left(T_{\mathrm{el}}\right) - G_{\mathrm{el-ph}}\left(T_{\mathrm{el}} - T_{\mathrm{ph}}\right) + S(r,t) \tag{8a}$$

$$C_{\mathrm{ph}}\frac{\partial T_{\mathrm{ph}}}{\partial t} = \kappa_{\mathrm{ph}}\nabla^2\left(T_{\mathrm{ph}}\right) + G_{\mathrm{el-ph}}\left(T_{\mathrm{el}} - T_{\mathrm{ph}}\right), \tag{8b}$$

where T_{ph} and C_{ph} are the phonon temperature and heat capacity, respectively, and κ_{el} and κ_{ph} the electron and phonon thermal conductivities, respectively. $S(r,t)$ is the source, which describes the heat deposited into the electronic subsystem by the pump pulse, modelled with a Gaussian spatial and temporal shape with standard widths $\sigma_{\mathrm{S,r}}$ and $\sigma_{\mathrm{S,t}}$, respectively. It assumes that the hot electrons are thermalized among each other immediately after excitation.

After the electrons and phonons have equilibrated, $T_{\mathrm{el}} \approx T_{\mathrm{ph}} = T$, and Eq. (8) can be simplified as

$$\left(C_{\mathrm{el}} + C_{\mathrm{ph}}\right)\frac{\partial T}{\partial t} = \left(\kappa_{\mathrm{el}} + \kappa_{\mathrm{ph}}\right)\nabla^2 T, \tag{9}$$

so the effective thermal diffusivity is

$$D_{\mathrm{eff}} = \frac{\kappa_{\mathrm{el}} + \kappa_{\mathrm{ph}}}{C_{\mathrm{el}} + C_{\mathrm{ph}}}. \tag{10}$$

Because the electron-phonon coupling time ($\tau_{\mathrm{el-ph}}$) is much shorter than our measurement resolution ($\sim$ 50 ps), we do not resolve the electron-phonon equilibration and measure the effective diffusivity D_{eff}.

9 Electronic thermal diffusivity and conductivity

To find the relation between the electrical σ and electronic heat κ conductivities, we use the model for free electrons in a Fermi gas, schematically illustrated in Fig. 3c in the main text. The electrical conductivity is related to the carrier density n and mobility $\mu = \frac{e\tau}{m}$, where e is the electron charge, τ the electron scattering time and m the effective mass. In a metal, such as $Ti_3C_2T_x$ MXene, the mobility is related to the electron diffusivity D by the Einstein relation for a Fermi gas [9]

$$D = \frac{2}{3}\frac{E_F \mu}{e}, \tag{11}$$

where $E_F = \frac{\hbar^2}{2m}\left(3\pi^2 n\right)^{2/3}$ is the Fermi level [10] and n is the electron density. Then, the electron thermal conductivity can be calculated as

$$\kappa_{el} = C_{el} D, \tag{12}$$

where the electronic heat capacity C_{el} is given by [10]

$$C_{el} = \frac{\pi^2}{2} n k_B^2 \frac{T}{E_F}, \tag{13}$$

where k_B is Boltzmann's constant and T the temperature. We recall the relation between electron mobility and conductivity:

$$\mu = \frac{\sigma}{ne}. \tag{14}$$

Inserting Eqs. 11 and (13-14) into Eq. 12 gives

$$\kappa_{el} = \frac{\pi^2}{3}\frac{k_B^2}{e^2}\sigma T, \tag{15}$$

which reproduces the Wiedemann-Franz law, and establishes the connection between the "macroscopic" parameters thermal conductivity and charge conductivity (Eq. 15 and Fig. 3b of the main text) with the "microscopic" parameters thermal diffusivity and charge mobility (Eq. 5 and Fig. 3a of the main text).

To calculate these quantities for $Ti_3C_2T_x$ MXene, we use the values from; [8] $n = 1.2 \times 10^{21}\,\mathrm{cm^{-3}}$, $\sigma = 1 \times 10^6\,\mathrm{Sm^{-1}}$ and $m = 0.28 m_e$. Given the negligible dispersion in the THz range, this THz conductivity represents the DC conductivity. Inserting these values into 11-15, $C_{el} = 1.7 \times 10^3\,\mathrm{Jm^{-3}K^{-1}}$, $\kappa_{el} = 7.3\,\mathrm{Wm^{-2}K^{-1}}$ and we obtain $D_{el,hot} = \kappa_{el}/C_{el} = 42\,\mathrm{cm^2s^{-1}}$. This is the heat carried by the electrons, and $D_{el,hot}$ is the diffusivity of the electronic heat, before thermalization with the phonons. At longer timescales, when the electrons are thermalized with the phonons, the thermal diffusivity due to electronic heat conduction requires using the total (electron + phonon) heat capacity C_V,

$$D_{el,th} = \frac{\kappa_{el}}{C_V}, \tag{16}$$

which results in $D_{el,th} \approx 0.025\,\mathrm{cm^2s^{-1}}$. Here, we used the experimentally determined heat capacity C_V from Ref. [7]

10 Volumetric Heat Capacity

The thermal conductivity κ and the thermal diffusivity D are related by

$$\kappa = C_V D, \tag{17}$$

where C_V is the the volumetric heat capacity, which should be given in units of $\mathrm{J\,m^{-3}\,K^{-1}}$. Ref.[16] reports the specific heat $C_p = 142\,\mathrm{J\,mol^{-1}\,K^{-1}}$. This related to C_V by

$$C_V = C_p \frac{\rho}{M}, \tag{18}$$

where $\rho = 4.12\,\mathrm{g\,cm^{-3}}$[17] is the density of $Ti_3C_2T_x$ MXene and $M = 185\,\mathrm{g\,mol^{-1}}$ the molar mass of Ti_3C_2OH, which is assumed by Ref.[16] This gives a volumetric heat capacity of $C_V = 3.16 \times 10^6\,\mathrm{J\,m^{-3}\,K^{-1}}$. This value is similar to the value reported by Ref.,[7] given the variation in surface termination that is expected due to slight differences in fabrication methods.

11 Heat Capacity Calculation

The thermal conductivity κ is proportional to the thermal diffusivity D, with the proportionality constant C_V, the volumetric heat capacity. Reference[16] reports the specific heat $C_p = 142\,\mathrm{J\,mol^{-1}K^{-1}}$, which is converted to C_V using the density $\rho = 4.12\,\mathrm{g\,cm^{-3}}$[17] and molar mass $M = 168\,\mathrm{g\,mol^{-1}}$, $C_V = C_p \frac{\rho}{M}$

References

[1] Block, A.: Quantifying nanoscale carrier diffusion with ultrafast optical and photocurrent microscopy. PhD thesis, Universitat Politècnica de Catalunya (September 2019). https://doi.org/10.5821/dissertation-2117-176428

[2] Suzaki, Y., Tachibana, A.: Measurement of the μm sized radius of Gaussian laser beam using the scanning knife-edge. Applied Optics **14**(12), 2809–2810 (1975) https://doi.org/10.1364/AO.14.002809

[3] Ramsden, H., van Hemert, M. C.. Autofocus. Eindhoven University of Technology (2025) https://github.com/0Hughman0/autofocus

[4] Maleski, K., Shuck, C.E., Fafarman, A.T., Gogotsi, Y.: The Broad Chromatic Range of Two-Dimensional Transition Metal Carbides. Advanced Optical Materials **9**(4), 2001563 (2021) https://doi.org/10.1002/adom.202001563

[5] Leung, P.T.: Surface-enhanced Raman scattering at elevated temperatures. Physical Review B **53**(19), 12659–12662 (1996) https://doi.org/10.1103/PhysRevB.53.12659

[6] Wang, T., Yao, C., Gao, R., Holicky, M., Hu, B., Liu, S., Wu, S., Kim, H., Ning, H., Torrisi, F., Bakulin, A.A.: Ultrafast Carrier and Lattice Cooling in Ti_2CT_x MXene Thin Films. Nano Letters **24**(51), 16333–16341 (2024) https://doi.org/10.1021/acs.nanolett.4c04583

[7] Tornero Gasca, E., Liu, B., Ippolito, S., van Hemert, M. C., Ramsden, H., David Saleta Reig, Schmidt, A.J., Ramos Jimenez, M., Klinar, K., Gogotsi, Y., Tielrooij, K.-J., Muñoz Rojo, M.: Temperature-dependent and thickness-independent out-of-plane heat transport of two-dimensional $Ti_3C_2T_x$ MXene flakes. Small, Under review (2026)

[8] Zheng, W., Ramsden, H., Ippolito, S., van Hemert, M., Zhang, D., Zhang, T., Li, D., Wen, G., Geuchies, J., Yu, M., Feng, X., Gogotsi, Y., Tielrooij, K.-J., Wang, H.I.: Photothermal effects control ultrafast charge transport in titanium carbide MXenes. Nature Communications **17**, 1201 (2026) https://doi.org/10.1038/s41467-026-68831-4

[9] Castner, T.G.: Einstein relations near the metal-insulator transition. Physical Review B **55**(7), 4003–4006 (1997) https://doi.org/10.1103/PhysRevB.55.4003

[10] Kittel, C.: Chapter 6: Free Electron Fermi Gas. In: Introduction to Solid State Physics, 8th ed edn., pp. 133–159. Wiley, Hoboken, NJ (2005)

[11] Downes, M., Shuck, C.E., McBride, B., Busa, J., Gogotsi, Y.: Comprehensive synthesis of $Ti_3C_2T_x$ from MAX phase to MXene. Nature Protocols **19**(6), 1807–1834 (2024) https://doi.org/10.1038/s41596-024-00969-1

[12] Block, A., Liebel, M., Yu, R., Spector, M., Sivan, Y., García de Abajo, F.J., van Hulst, N.F.: Tracking ultrafast hot-electron diffusion in space and time by ultrafast thermomodulation microscopy. Science Advances **5**(5), 8965 (2019) https://doi.org/10.1126/sciadv.aav8965

[13] Varghese, S., Mehew, J.D., Block, A., Reig, D.S., Woźniak, P., Farris, R., Zanolli, Z., Ordejón, P., Verstraete, M.J., Van Hulst, N.F., Tielrooij, K.-J.: A pre-time-zero spatiotemporal microscopy technique for the ultrasensitive determination of the thermal diffusivity of thin films. Review of Scientific Instruments **94**(3), 034903 (2023) https://doi.org/10.1063/5.0102855

[14] Block, A., Yu, R., Un, I.-W., Varghese, S., Liebel, M., Van Hulst, N.F., Fan, S., Tielrooij, K.-J., Sivan, Y.: Observation of Negative Effective Thermal Diffusion in Gold Films. ACS Photonics, 2–01916 (2023) https://doi.org/10.1021/acsphotonics.2c01916

[15] Anayee, M., Wang, R., Downes, M., Ippolito, S., Gogotsi, Y., Layer-by-layer mechanism of the MAX phase to MXene transformation. Matter **8**(6), 102092 (2025) https://doi.org/10.1016/j.matt.2025.102092

[16] Srivatsa, S., Tokarz, W., Przewoźnik, J., Strączek, T., Grabowski, K., Rutkowski, P., Uhl, T., Kulawik, J., Kata, D., Madej, D., Lis, J., Kapusta, C.: Temperature Evolution of Composition, Thermal, Electrical and Magnetic Properties of $Ti_3C_2T_x$-MXene. Materials **17**(10), 2199 (2024) https://doi.org/10.3390/ma17102199

[17] Sigma-Aldrich: Titanium Carbide ($Ti_3C_2T_x$-MXene) Few layer MXene. https://www.sigmaaldrich.com/NL/en/product/aldrich/924962
